# Enhanced Efficacy of Chemotherapy by Addition of Immune Checkpoint Inhibitors in Stage IV Large Cell Neuroendocrine Carcinoma of the Lung: A Real-World Analysis

**DOI:** 10.7150/jca.87052

**Published:** 2023-09-25

**Authors:** Lingbin Meng, Biwei Cao, Rui Ji, Dung-Tsa Chen, Damian A. Laber, Michael Shafique

**Affiliations:** 1 Department of Hematology and Oncology, H. Lee Moffitt Cancer Center & Research Institute, University of South Florida, Tampa, FL, USA.; 2 Division of Medical Oncology, Department of Internal Medicine, The Ohio State University Comprehensive Cancer Center, Columbus, OH, USA.; 3 Department of Biostatistics and Bioinformatics, H. Lee Moffitt Cancer Center & Research Institute, Tampa, FL, USA.; 4 Department of Satellite and Community Oncology, H. Lee Moffitt Cancer Center & Research Institute, University of South Florida, Tampa, FL, USA.; 5 Department of Thoracic Oncology, H. Lee Moffitt Cancer Center & Research Institute, University of South Florida, Tampa, FL, USA.

**Keywords:** Immunotherapy, Chemotherapy, immune checkpoint inhibitors, LCNEC, overall survival

## Abstract

**Background:** Large Cell Neuroendocrine Carcinoma (LCNEC) is a high-grade malignancy with limited treatment options. Despite promising results of immunotherapy in non-small cell and small cell lung cancers, its benefit in LCNEC remains elusive.

**Methods:** We included 24 patients diagnosed with stage IV LCNEC from the Moffitt Cancer Center database who received systemic therapy between January 2016 and May 2021. Group A comprised patients who received first-line CT and ICI (anti-PD-1 or anti-PD-L1 therapy for ICI, n = 11), and Group B received first-line CT only (n = 13). The collected data encompassed overall survival (OS), progression-free survival (PFS), objective response rate (ORR), disease control rate (DCR), and toxicities since treatment initiation.

**Results:** Kaplan-Meier survival analysis revealed median OS was 56 weeks (95%CI = 22.2-89.8) and 28 weeks (95% CI=16.3-39.7) in groups A and B, respectively. Log-rank test showed the difference was statistically significant (*p*=0.029). Median PFS was 32 weeks (95%CI=14.7-49.3) in group A and 20 weeks (95% CI=13.8-26.2) in groups B, but the difference was not statistically significant (*p*= 0.136). Univariate Cox analysis confirmed that the addition of ICI to CT significantly improved OS in patients with stage IV LCNEC (HR=0.35, 95% CI=0.13-0.95, *p* = 0.039). The ORR (63.6% vs 45.4%, *p*= 0.670) and DCR (81.8% vs 63.6%, *p*= 0.635) tended to be higher in group A than in group B but the difference was not statistically significant. Importantly, the combined treatment demonstrated a satisfactory safety profile, with only two patients reporting grade 2 or higher adverse events.

**Conclusions:** Our results suggest that the combination of immunotherapy with chemotherapy holds potential for improving outcomes in stage IV LCNEC. Despite the retrospective nature and limited sample size of our study, these preliminary findings provide a valuable insight into the potential of immunotherapy in LCNEC treatment and encourage further research through larger, prospective trials.

## Introduction

Large cell neuroendocrine carcinoma of the lung (LCNEC) is a rare and aggressive malignancy, accounting for less than 3% of all lung cancer cases 1, 2. According to the 2015 World Health Organization (WHO) classification, LCNEC is defined by large cells with abundant cytoplasm, high mitotic rate, extensive necrosis, and neuroendocrine differentiation 3. It shares characteristics of both small cell lung cancer (SCLC) and non-small cell lung cancer (NSCLC). For instance, LCNEC demonstrates aggressive behavior, high recurrence rates, and metastatic patterns resembling SCLC 4-6, while its stage distribution at presentation is akin to that of NSCLC 6. Molecularly, LCNEC can be further categorized into SCLC-like subtypes, characterized by co-mutations in TP53 and RB1 genes, and NSCLC-like subtypes, which lack TP53 or RB1 mutations but exhibit mutations in KRAS, STK11, or KEAP1 genes 7.

Owning to its rarity, the optimal systemic therapy for advanced LCNEC remains elusive, and treatment approaches are extrapolated from SCLC (i.e., platinum plus etoposide) or NSCLC (i.e., platinum plus taxane) regimens. The superiority of either regimen remains a matter of debate. Some studies have reported better survival outcomes for stage IV LCNEC patients treated with SCLC regimens compared to NSCLC regimens 8-10 , while other research has demonstrated more favorable results for metastatic LCNEC patients treated with NSCLC chemotherapy regimens 11-13. Regardless of the chosen approach, none have shown satisfactory efficacy in treating LCNEC. For example, overall response rates in SCLC and NSCLC regimen groups were 73% and 50% (*p* = 0.19), with median progression-free survival (PFS) of 6.1 and 4.9 months (P = 0.41), and median overall survival (OS) of 16.5 vs. 9.2 months (*p* = 0.10), respectively 8. Beyond the realm of chemotherapy, exploratory endeavors have been undertaken to investigate the potential applicability of targeted therapy within the LCNEC paradigm 14-16. Nevertheless, the scarcity of LCNEC has precluded the establishment of any tangible clinical benefit associated with targeted therapy for this particular neoplasm. The circumscribed efficacies observed in existing treatment modalities highlight the urgent need for innovative treatment strategies to enhance the therapeutic outcomes for LCNEC patients.

Over the past decade, significant advancements in lung cancer treatment have been achieved through the development of immune checkpoint inhibitors (ICI). Several ICI have been approved as monotherapy or in combination with chemotherapy for various human cancer types. Pembrolizumab, nivolumab, atezolizumab, and durvalumab have received FDA approval for the treatment of advanced NSCLC 17-19. The combination of atezolizumab or durvalumab, monoclonal antibodies targeting programmed cell death ligand 1 (PD-L1), with first-line chemotherapy has led to improved OS in patients with extensive stage SCLC 20, 21. Although these agents are currently under investigation in early-stage SCLC and NSCLC settings, the efficacy of immunotherapy for LCNEC remains uncertain due to the disease's rarity and lack of prospective evidence. Most available data stem from small, retrospective case series and case reports 22-25, emphasizing the need for further research.

To address this knowledge gap, we conducted a real-world cohort study aimed at investigating the efficacy of adding ICIs to standard chemotherapy (CT) in the treatment of stage IV LCNEC patients. Our study evaluated multiple endpoints, including OS, PFS, objective response rate (ORR), and disease control rate (DCR).

## Patients and Methods

### Study Population and treatment

We evaluated all cases of de novo stage IV LCNEC diagnosed at Moffitt Cancer Center between January 2016 and May 2021. The diagnosis of LCNEC was conducted by pathologists at Moffitt Cancer Center using tissue biopsy, in accordance with the histopathological criteria outlined in the WHO classification. Immunohistochemistry was applied to verify neuroendocrine differentiation in all specimens. A total of 24 chemotherapy and immunotherapy-naive patients diagnosed with LCNEC received CT at Moffitt Cancer Center, with 11 of them also concurrently undergoing immunotherapy. In line with NCCN guidelines and taking into account the lack of contraindications for immunotherapy, such as autoimmune disease, physicians elected to administer ICI immunotherapy to these 11 patients, encompassing pembrolizumab and atezolizumab. All patients were subsequently categorized into Group A, which comprised patients who received first-line CT and ICI (n = 11), and Group B, which included those who received first-line CT only (n = 13). The CT consisted of an SCLC regimen encompassing etoposide in combination with carboplatin/cisplatin or carboplatin and irinotecan, and an NSCLC regimen involving paclitaxel, pemetrexed, gemcitabine, and carboplatin/cisplatin. The administered immunotherapy regimens included atezolizumab or pembrolizumab.

### Study Design and assessments

After obtaining approval from the institutional ethical review board, we conducted a retrospective review of patients' charts and hospital electronic medical records, collecting baseline demographic, clinical, pathological, and treatment characteristics. OS since the first treatment was recorded and compared between groups. PFS was defined as the time from the first chemotherapy administration until objective tumor progression or death from any cause, whichever occurred first. ORR was defined as the sum of partial and complete responses. Response was categorized as stable disease (SD), partial response (PR), complete response (CR), or progressive disease (PD). Tumor progression was assessed using the World Health Organization and Response Evaluation Criteria in Solid Tumors (RECIST). Furthermore, we examined the safety of ICI in the combination group. Adverse events were graded using the Common Terminology Criteria for Adverse Events, version 4.03 (CTCAE, v. 4.03).

### Statistical analysis

Baseline demographic, clinical, and pathological characteristics were assessed using Fisher's exact test. OS and PFS were analyzed using the Kaplan-Meier method and compared with the log-rank test. A univariate Cox proportional hazards model was employed to determine the association of clinical variables with overall survival. Response rates were compared using Fisher's exact test. In this study, P values were two-sided, and a P value of less than 0.05 was deemed statistically significant.

## Results

### Demographics and clinical characteristics of metastatic LCNEC patients

A total of twenty-four participants who fulfilled the inclusion criteria were incorporated into the analysis. The subjects were divided into Group A, which included patients who received first-line CT plus ICI (n = 11), and Group B, which consisted of those who received first-line CT only (n = 13). Both groups of LCNEC patients exhibited comparable baseline characteristics, providing a suitable foundation for further evaluation (Table 1). However, Group B appeared to have a younger median age (61 vs 70), superior Eastern Cooperative Oncology Group (ECOG) performance status (90.9% vs 61.5% in ECOG 0/1), a higher prevalence of brain metastases (63.6% vs 38.5%), and liver metastases (45.5% vs 15.4%), but these differences were not statistically significant.

### Improved OS and a trend of better PFS in metastatic LCNEC patients treated with CT plus ICI

**Figure 1:**
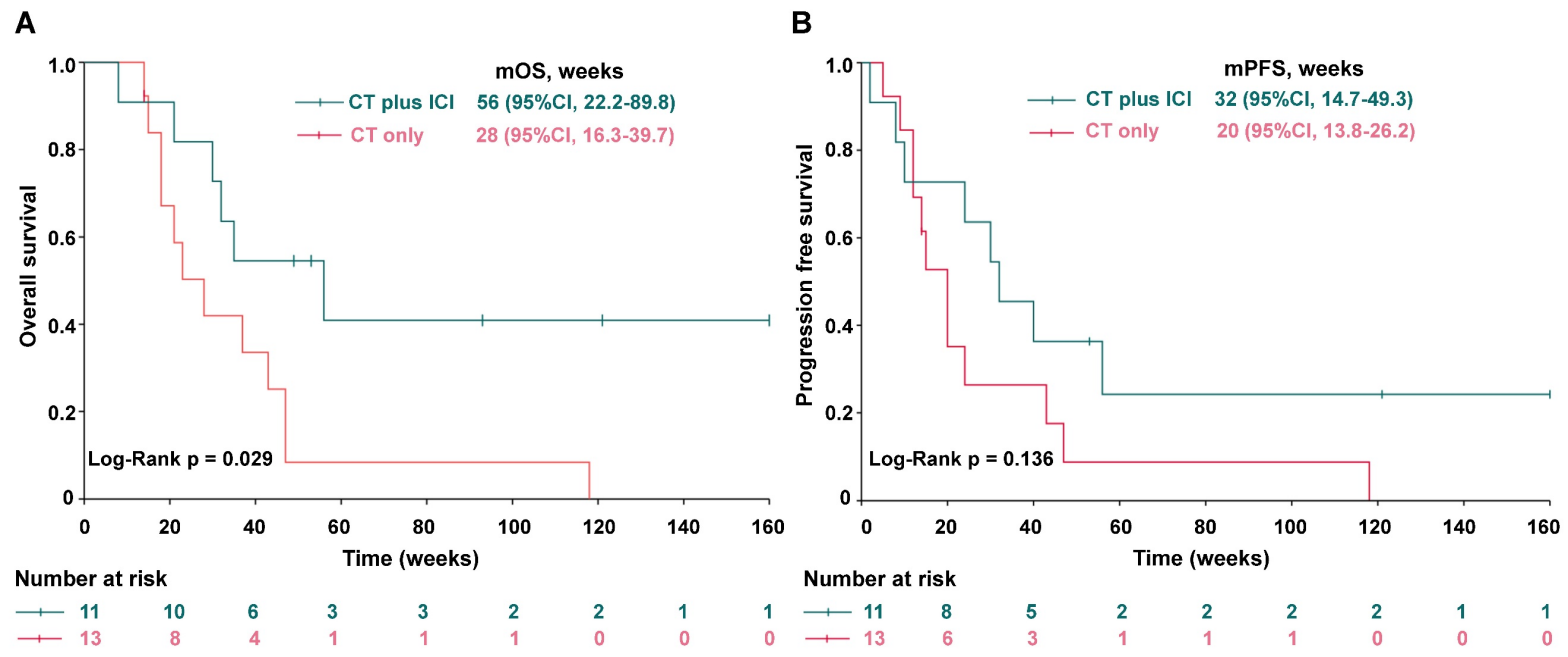
Improved overall survival (OS) and a trend of better progression free survival (PFS) in metastatic LCNEC patients treated with CT plus ICI. (A) OS and (B) PFS of stage IV LCNEC patients treated with CT only vs CT plus ICI.

Group A, comprising patients treated with CT plus ICI, demonstrated a significantly better median overall survival (mOS) compared to Group B, which received CT only (Figure 1). Group A had a mOS of 56 weeks (95% CI = 22.2-89.9) versus 38 weeks (95% CI = 16.3-39.7) in Group B (log-rank test, *p* = 0.029; Fig. 1A).

Additionally, Group A exhibited a trend of better median PFS (mPFS), with a mPFS of 32 weeks (95% CI, 17.4-49.3) compared to 20 weeks (95% CI, 13.8-26.2) in Group B. However, due to the limited sample size, the data did not reach statistical significance (log-rank test, *p* = 0.136; Fig. 1B).

In the univariate Cox regression analysis (Table 2), treatment with CT plus ICI was found to be significantly correlated with better OS (HR = 0.35; 95% CI = 0.13-0.95; *p* = 0.039). However, no significant correlation was observed between OS and age (HR = 1.04; 95% CI = 0.98-1.10; *p* = 0.163), gender (HR = 0.41; 95% CI = 0.15-1.10; *p* = 0.077), smoking history (HR = 23.47; 95% CI = 0.01-703.85; *p* = 0.433), ECOG status (HR = 2.28; 95% CI = 0.78-6.66; *p* = 0.132), chemotherapy regimen options (HR = 1.26; 95% CI = 0.48-3.34; *p* = 0.638), brain metastases (HR = 0.65; 95% CI = 0.25-1.65; *p* = 0.363), liver metastases (HR = 1.32; 95% CI = 0.49-3.59; *p* = 0.585), or bone metastases (HR = 0.82; 95% CI = 0.32-2.15; *p* = 0.693).

### A trend of higher ORR and DCR in metastatic LCNEC patients treated with CT plus ICI

**Figure 2:**
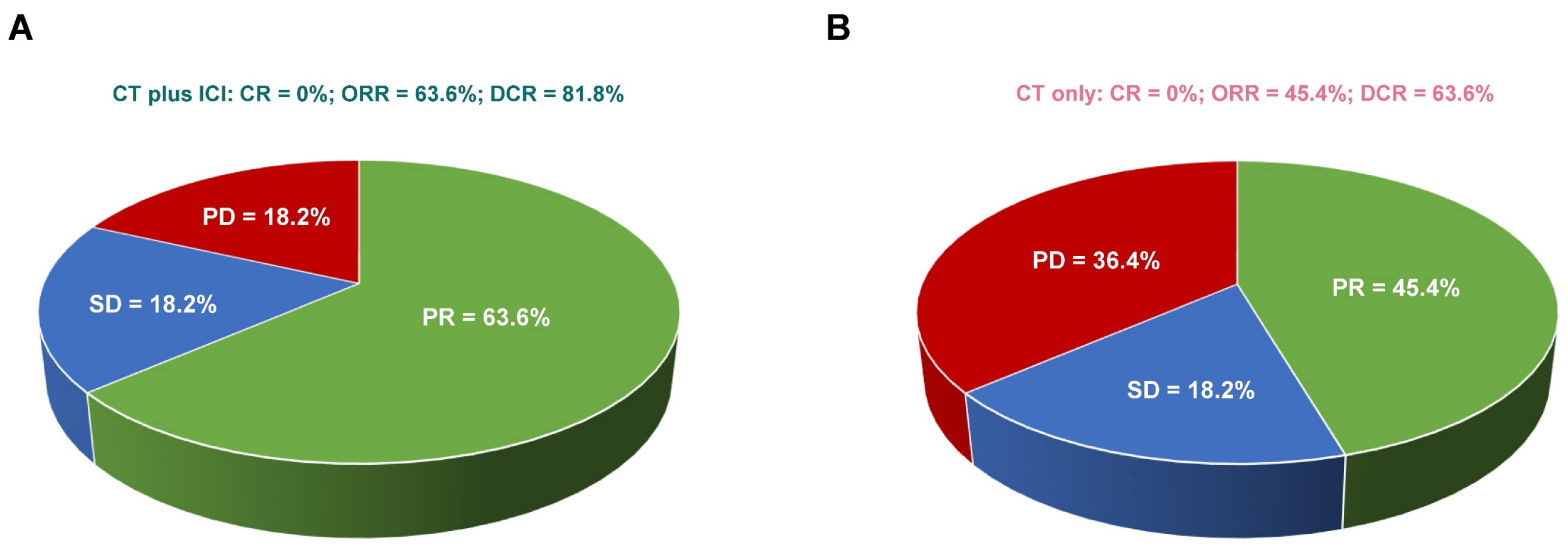
A trend of higher ORR (p = 0.670) and DCR (p = 0.635) in metastatic LCNEC patients treated with CT plus ICI. Comparison of response rates in stage IV LCNEC patients receiving CT plus ICI (A) versus CT only (B). CR, complete response; PR, partial response; SD, stable disease; ORR, objective response rate; DCR, disease control rate.

Eleven patients from each group underwent adequate computer tomography (CT)/positron emission computer tomography (PET-CT) scans for radiological assessment. As illustrated in Figure 2, none of the patients achieved a complete response (CR). In patients treated with CT plus ICI, seven (63.6%) had partial response (PR), two (18.2%) had stable disease (SD), and two (18.2%) had disease progression (PD). In patients treated with CT only, five (45.4%) had PR, two (18.2%) had SD, and four (36.4%) had PD. Consequently, patients treated with CT plus ICI displayed a trend of higher ORR (CR+PR: 63.6% vs 45.4%, *p*=0.670) and DCR (ORR+SD: 81.8% vs 63.6%, *p*=0.635) compared to those treated with CT only, but the difference was not statistically significant (Fisher's exact test).

### Safety of ICI in metastatic LCNEC patients treated with CT and ICI

In the group of patients receiving CT plus ICI, two patients experienced grade 2 or higher immune-related adverse events. Specifically, one patient developed grade 2 autoimmune hemolytic anemia after receiving pembrolizumab, which was successfully managed with high-dose intravenous corticosteroids, intravenous immunoglobulin, and rituximab. Another patient developed grade 3 pneumonitis after receiving one dose of immunotherapy and subsequently developed acute respiratory failure, which unfortunately resulted in the patient's death. No other patients in this group experienced severe side effects, and none had to discontinue treatment due to adverse effects.

## Discussion

LCNEC is a rare and aggressive malignancy, and the optimal systemic therapy for advanced LCNEC has demonstrated limited efficacy. Although immunotherapy has shown promising results in NSCLC 17-19 and SCLC 20, 21, its effectiveness in LCNEC remains uncertain due to the disease's rarity and lack of prospective evidence. In this retrospective cohort study, we examined the efficacy of incorporating ICI with standard CT for treating patients with stage IV LCNEC. Our findings revealed a significant improvement in OS, with trends towards enhanced PFS, ORR and DCR upon adding ICI to standard CT. These results offer valuable insights into the potential benefits of combining immunotherapy and chemotherapy for managing stage IV LCNEC.

Several recent publications have attempted to assess the effectiveness of immunotherapy in advanced LCNEC. For instance, Agar et al. evaluated the efficacy of nivolumab in 51 stage III and IV LCNEC patients, including 17 who received nivolumab as second-line treatment or beyond 22. Their results did not indicate any difference in the efficacy between nivolumab and conventional treatment groups. Similarly, Sherman et al. assessed the activity and safety of ICIs in 37 patients with stage III and IV LCNEC, with 23 treated with immunotherapy, and did not observe any superiority of immunotherapy over conventional therapy 24. Conversely, another retrospective analysis investigated the outcomes of ICIs in 125 stage III and IV LCNEC patients, with 41 receiving ICI as any treatment line, and reported a significantly positive impact of ICI on OS in advanced LCNEC 25. These studies included both stage III and IV LCNEC patients who might receive immunotherapy at any treatment stage, possibly introducing confounding biases concerning survival data due to differences in staging and treatment lines between groups. Recently, Komiya et al. used data from the National Cancer Database (NCDB) to evaluate the impact of ICIs on the OS of stage IV LCNEC patients, revealing an association between ICI use and improved OS 23. However, this study lacked data on PFS, response rates, and toxicity profiles. Despite its limited sample size, our study exclusively included stage IV LCNEC patients naïve to any systemic therapy, thereby minimizing confounding and selection biases. In addition to demonstrating a significant impact on OS from ICI, we observed trends of better PFS, ORR, and DCR in stage IV LCNEC patients treated with CT plus ICI.

The optimal first-line chemotherapy regimen for metastatic LCNEC continues to be a subject of debate, with some studies supporting the use of SCLC regimens 8-10 while others recommending NSCLC regimens 11-13. For stage IV LCNEC, platinum-based chemotherapy regimens commonly employed for SCLC typically produce higher ORRs (37-52%) than those used for NSCLC (12-50%) 8. However, these responses are generally short-lived, with a median PFS of 4.6-6.1 months, and the OS remains poor, with a median of 10.2-11.1 months 8, 26. One study also discovered that the OS of LCNEC patients treated with an NSCLC-based regimen was significantly longer than those treated with an SCLC-based regimen, with median survival times of 8.5 and 6.7 months, respectively 11. Our study did not determine the superiority of one regimen over the other, as Table 2 revealed no correlation between chemotherapy regimen choices and OS (HR = 1.26; 95% CI = 0.48-3.34; *p* = 0.638). Owing to the limited sample size, larger-scale investigations are needed to identify the most effective chemotherapy regimen to combine with immunotherapy for stage IV LCNEC patients.

Retrospective studies have estimated the rate of PD-L1-positive LCNEC to be between 10% and 27% 27, 28. In our study, PD-L1 levels were recorded in 9 patients treated with CT plus ICI and 6 in patients treated with CT only. One patient from each group exhibited positive PD-L1 expression, with 90% positive staining in the CT plus ICI group and 20% positive staining in the CT-only group. Consequently, 13.3% (2/15) of stage IV LCNEC patients were PD-L1 positive, which aligns with the reported values in the literature. Given that only one PD-L1 positive patient was present in the group treated with CT plus ICI, it suggests that the efficacy of ICI in LCNEC may be independent of PD-L1 expression levels. This observation is consistent with previous findings 25, 29. The independence of ICI efficacy from PD-L1 expression levels may be attributed to the complex nature of the tumor microenvironment, where factors such as tumor-infiltrating immune cells, alternative immune checkpoint molecules, and other immune modulators may play a role in determining the response to immunotherapy 30, 31. As a result, immunotherapy utilizing checkpoint inhibitors may serve as an effective treatment option for patients with metastatic LCNEC, irrespective of PD-L1 expression status. Further research is necessary to elucidate the mechanisms underlying this phenomenon and to identify additional biomarkers that may predict response to ICI in this patient population.

The safety profile for patients treated with the combination of CT and ICI appears to be favorable for the majority. In this group, only two patients experienced grade 2 or higher immune-related adverse events, with one developing grade 2 automimic hemolytic anemia and another experiencing grade 3 pneumonitis. Notably, no other patients in this group encountered severe adverse effects, and none required treatment discontinuation due to adverse events. These findings suggest that the combination of immunotherapy with chemotherapy may be generally well-tolerated in patients with metastatic LCNEC, although careful monitoring and management of potential immune-related adverse events is essential.

The limitations of this study should be acknowledged when interpreting the results. First, the retrospective nature of the study inherently carries the risk of confounding biases and may limit the generalizability of the findings. Second, the small sample size reduces the statistical power of the study, which may have contributed to the lack of significance in some of the observed trends, such as the PFS, ORR and DCR. Additionally, the CT plus ICI group seemed to have a younger median age, superior performance status, a higher propensity to receive NSCLC-based chemotherapy, and a higher incidence of brain and liver metastases, all of which could potentially contribute to the enhanced OS observed in this group (Table 1). Despite the Fisher's exact test negating correlations between immunotherapy and these factors, and even though univariate Cox analysis ruled out their impact on OS, a comprehensive multivariate analysis for OS should be conducted to account for these factors. However, the limited sample size precluded the feasibility of performing such multivariate analyses for OS. Therefore, these discernible discrepancies underscore the potential for residual confounding, underscoring the necessity for larger, prospective studies with well-balanced patient characteristics to corroborate these findings.

In conclusion, our study demonstrates the potential benefits of adding ICI to standard CT in the treatment of stage IV LCNEC, with a significant improvement in OS and trends towards improved PFS, ORR, and DCR. These findings highlight the need for further research to optimize treatment strategies for patients with metastatic LCNEC, including the investigation of molecular characteristics and the determination of the most effective chemotherapy regimens in combination with immunotherapy.

## Figures and Tables

**Table 1:** Demographics and clinical characteristics of patients with stage IV LCNEC stratified by treatment options: CT only vs CT plus ICI.

	Pts treated with CT only (n=13)	Pts treated with CT plus ICI (n=11)	P value	All Patients (n=24)
**Age** (Median, IQR), years	70 (65-73)	61 (58-64.5)	0.082	65 (58.75-70.25)
**Gender**, n (%)			0.679	
Male	8 (61.5)	8 (72.7)		16 (66.7)
Female	5 (38.5)	3 (27.3)		8 (33.3)
**Smoking history**, n (%)			0.458	
Smoker	13 (100)	10 (90.9)		23 (95.8)
Never smoker	0 (0)	1 (9.1)		1 (4.2)
**ECOG**, n (%)			0.166	
0/1	8 (61.5)	10 (90.9)		18 (75)
2/3	5 (38.5)	1 (9.1)		6 (25)
**Chemotherapy**, n (%)			0.423	
SCLC-based	6 (46.2)	3 (27.3)		9 (37.5)
NSCLC-based	7 (53.8)	8 (72.7)		15 (62.5)
**Brain metastases**, n (%)			0.414	
Yes	5 (38.5)	7 (63.6)		12 (50)
No	8 (61.5)	4 (36.4)		12 (50)
**Liver metastases**, n (%)			0.182	
Yes	2 (15.4)	5 (45.5)		7 (29.2)
No	11 (84.6)	6 (54.5)		17 (70.8)
**Bone metastases**, n (%)			0.697	
Yes	6 (46.2)	4 (36.4)		10 (41.7)
No	7 (53.8)	7 (63.6)		14 (58.3)

**Table 2:** Univariate Cox regression analyses of clinical variables for predicting overall survival.

Variable	HR (95%CI)	P value
**Treatment:** CT plus ICI vs CT only	0.35 (0.13 - 0.95)	**0.039**
**Age**	1.04 (0.98 - 1.10)	0.163
**Gender:** male vs female	0.41 (0.15 - 1.10)	0.077
**Smoking history:** yes vs no	23.47 (0.01 - 703.85)	0.433
**ECOG:** 2/3 vs 0/1	2.28 (0.78 - 6.66)	0.132
**Chemotherapy:** SCLC vs NSCLC	1.26 (0.48 - 3.34)	0.638
**Brain metastases:** yes vs no	0.65 (0.25 - 1.65)	0.363
**Liver metastases:** yes vs no	1.32 (0.49 - 3.59)	0.585
**Bone metastases:** yes vs no	0.82 (0.32 - 2.15)	0.693
